# Green Synthesis of Ag/TiO_2_ Nanoparticles: Properties and Antibacterial Potential for Environmental Applications

**DOI:** 10.1021/acsomega.5c12007

**Published:** 2026-04-28

**Authors:** Caroline Zarzzeka, Jonas Goldoni, Filomena Marafon, Gilnei Bruno da Silva, Daiane Manica, Maria Eduarda Kounaris Fuziki, Fernando Manzotti de Souza, Margarete Dulce Bagatini, Leda Maria Saragiotto Colpini

**Affiliations:** † 28122 Federal University of Parana (UFPR), Palotina Setor—Postgraduate Program in Technology and Environmental Engineering, Pioneiro Street, 2153, 85950-000 Palotina, Brazil; ‡ 232192 Federal University of Fronteira Sul (UFFS), Chapecó Campus, SC 484 Highway, Km 02, 89815-899 Chapecó, Brazil; § State University of Santa Catarina (UDESC), Multicentric Postgraduate Program in Biochemistry and Molecular Biology, Luiz de Camões Avenue, 2090, 88520-000, Lages, Brazil; ∥ Federal University of Santa Catarina (UFSC), Postgraduate Program in Biochemistry, University Campus Rector João David Ferreira Lima, 88040-900 Florianópolis, Brazil; ⊥ Federal University Technology of Parana (UTFPR), Ponta Grossa Campus, Monteiro Lobato Avenue, Km 04, 84016-210 Ponta Grossa, Brazil; # Federal Institute of Santa Catarina (IFSC), Bertonni Street, 121, 89254-430 Jaraguá do Sul, Brazil

## Abstract

The spread of bacteria resistant to multiple drugs has required the development of new strategies for treating infections. Light-activated materials are emerging as a promising alternative, as they can inactivate bacteria by generating reactive oxygen species (ROS), without inducing resistance. Based on this search for new materials, silver (Ag) catalysts (2% and 10%) supported on titanium dioxide (TiO_2_) were synthesized via modified sol–gel synthesis, utilizing tapioca as a sustainable gelling agent. The Ag/TiO_2_ catalysts (2AgT/V and 10AgT/V) characterized by spectra in the infrared region showed characteristic titanium bands, asymmetric Ti–Ag–O vibration and Ag-TiO_2_ bonding, confirming the deposition of Ag on the support. X-ray diffraction identified characteristic peaks of metallic Ag and the anatase phase for the AgT/V catalysts, representing organized crystalline phases. Antibacterial assays demonstrated the high efficiency of the 10AgT/V catalyst, achieving a 98% reduction in *Escherichia coli* growth in 15 min in the absence of light, suggesting an intrinsic antimicrobial effect of Ag. Photoactivation of the 10AgT/V catalyst under black light reduced *E. coli* and *Staphylococcus aureus* by 85% and 84%, respectively, in 15 min. The production of ROS, confirmed in the detection test, along with the intrinsic action of Ag, explains the high performance of these catalysts. These results demonstrate the potential of AgT/V catalysts for the development of antimicrobial coatings, contributing to the prevention and control of infections in various environments.

## Graphical abstract

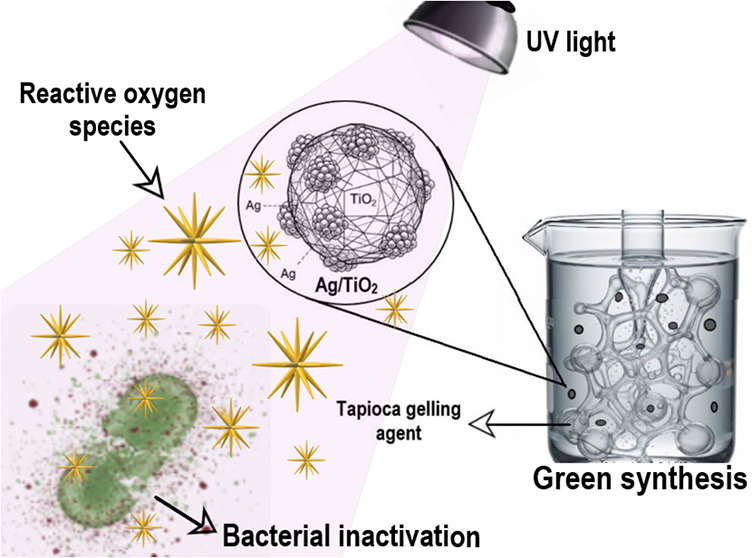

## 1 Introduction

Photocatalysis is an oxidation technique with potential in the degradation of organic pollutants, water treatment and the inactivation of microorganisms, and is environmentally friendly and safe. It occurs when a semiconductor, such as titanium dioxide (TiO_2_), is illuminated by a photon causing the excitation of electrons (e^–^) belonging to the valence band, which move to the conduction band where they couple with gaps (h^+^), forming electron–lacuna pairs (e^–^/h^+^). Through redox processes, e^–^ and h^+^ generate powerful ROS on the surface of the semiconductor, which subsequently degrade impurities into carbon dioxide and water, and can also eliminate bacteria.^1,2^ Examples of pathogens include *Staphylococcus aureus*, a Gram-positive bacterium, and *Escherichia coli*, a Gram-negative pathogen. These bacteria are transmitted through food, water and air,^3^ survive on various surfaces for long periods.^4,5^

TiO_2_ is a semiconductor that stands out in water purification systems, sterilization and self-cleaning coatings, thanks to its photoelectrochemical, photoinduced, superhydrophilic characteristics and does not produce harmful by products.^6,7^ However, this oxide has some limitations, such as the wide band energy range, the wavelength region restricted to the ultraviolet, the low quantum efficiency and the fast recombination rate of the e^–^/h^+^ pairs.^8,9^ Doping with a transition metal, such as Ag, improves the aforementioned limitations of TiO_2_, as the metal acts as an e^–^ capture trap, alters the absorption of visible light, optimizes plasmon resonance and increases the photocatalytic action.^9,10^ In addition, the antimicrobial characteristics of TiO_2_ are improved with Ag doping, thanks to the high surface-to-volume ratio, wide therapeutic range, greater stability and remarkable antibacterial activities.^11,12^

The antibacterial efficiency of Ag/TiO_2_ is high, because during the exposure time both TiO_2_ and Ag^+^ ions are released, resulting in the interruption of the metabolic activity of the bacterial cell membrane. Ag^+^ ions facilitate binding affinity to the surfaces of bacterial membranes, which are negatively charged.^13,14^ Furthermore, Ag nanoparticles on the surface of cells trigger structural rearrangement of the membrane, thus favoring the entry of ROS into the bacteria, causing DNA damage. Nanomaterials containing Ag can act as multifunctional agents, exerting antibacterial and photodegradation properties, as is the case with Ag/TiO_2_. They can act on more than one problem, such as organic pollutants and pathogens, making the use of these catalysts more efficient and promising.^15^

The sol–gel method has aroused interest due to its simplicity, reproducibility and mild conditions.^16^ To improve this method, a variety of complexing agents have been used to reduce production costs.^17^ For example, low-cost commercial starch, such as tapioca, is used in the synthesis of particles via the modified sol–gel route, which represents a breakthrough in the synthesis of semiconductors and is also considered green synthesis. This technique is less expensive, generates environmentally sustainable by products and reduces the toxicity of the synthesized material. It also uses microorganisms, vitamins, enzymes, amino acids, plant extracts or plants during synthesis.^18,19^

Among plants, *Manihot esculenta* or cassava contains a high carbohydrate content, around 38%, making it a valuable resource in green synthesis.^20^ It also has secondary metabolites, including antioxidant compounds such as terpenes and β-carotene, as well as other biomolecules, ensuring particle stability and minimizing agglomeration.^21,22^ Tapioca, made from cassava starch, is a natural polymer that is abundant in nature, renewable, biodegradable and easily stored. It has a uniform constitution with a low-order crystalline structure, which contains an incomplete bond between two glucose molecules, which provides affinity with metal ions.^23,24^ The use of tapioca as a chelating agent in the synthesis of oxides has enormous potential, as it makes production more accessible, simple, economical, cheap and uses low temperatures, as well as increasing the flexibility and strength of composite materials.^25,26^

This study aims to synthesize mixed Ag/TiO_2_ oxides (2 and 10% w/w% Ag) using tapioca in the modified sol–gel process. As well as evaluating their physicochemical characterizations and their antibacterial activity, under clear light, black light and absence of light.

## 2 Materials and Methods

### 2.1 Materials

Commercial Titanium dioxide (TiO_2_, Êxodo Científica) and silver nitrate (AgNO_3_, Alphatec) were used, both analytical grade and without any additional purification. The water used in the synthesis was of Milli-Qplus quality with an approximate resistivity of 18 MΩ·cm. The antimicrobial activity of the catalysts was studied using the bacteria *Escherichia coli* (ATCC 25922) and *Staphylococcus aureus* (ATCC 25923).

### 2.2 Modified Sol–Gel Synthesis/Green Synthesis

To obtain mixed oxides synthesized by the modified sol–gel route, known as green synthesis and using tapioca as a gelling agent, adaptations were made to the methodologies.^23,27^ To use TiO_2_ as a support in the preparation of mixed oxides, it was dried in an oven for 21 h at 120 °C. Five g of tapioca was weighed and mixed with 150 mL of water (solution A) and kept stirring. A previously prepared solution of TiO_2_ and AgNO_3_ (solution B) in 50 mL of water was slowly added to this solution A. The mixture was then heated at 80 °C for 1 h to form a viscous, homogeneous solution. The final solution was placed in an oven for 24 h at 100 °C and then calcined, with a heating ramp, at 400 °C for 5 h. In order to study the effect of Ag concentration on the properties of the materials, the mixed oxides described were obtained containing proportions of Ag varying between 2% and 10% by mass, 0.9638 and 5.2477 g, respectively. The TiO_2_ catalysts (commercial sample), 2% Ag/TiO_2_/Green synthesis and 10% Ag/TiO_2_/Green synthesis are represented as T, 2AgT/V and 10AgT/V. Figure 1 shows the modified sol–gel/green synthesis process of the mixed oxides.

**Figure 1:**
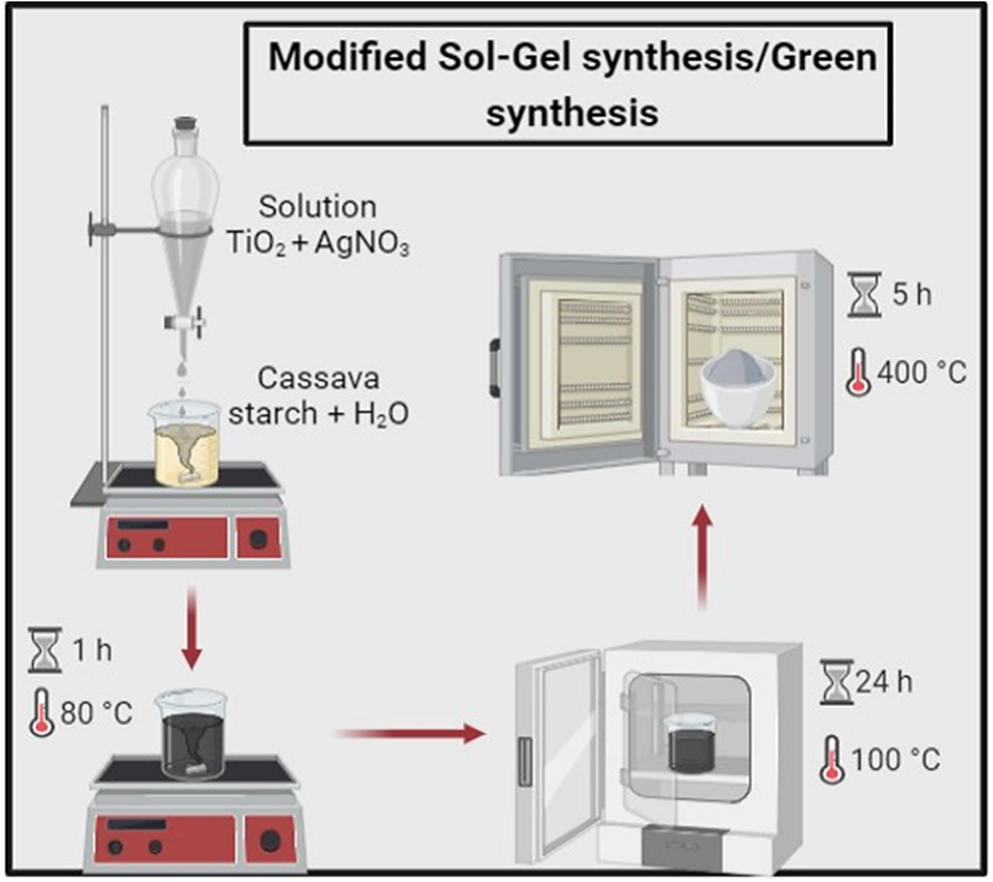
Modified sol–gel synthesis/green synthesis of mixed oxides. Illustration of the steps to synthesize Ag/TiO_2_ using a modified sol–gel route using cassava starch as a gelling agent, also known as green synthesis.

### 2.3 Characterization of the Catalysts

Spectra in the infrared region were analyzed from pellets of the materials dispersed in KBr, covering the 4000 to 500 cm^–1^ range, using a PerkinElmer FT-IR spectrophotometer, model Frontier. Confirmation of the crystal structure of the catalysts was obtained from X-ray diffraction (XRD) patterns using Rigaku’s Miniflex 600, with Cu kα radiation (λ = 0.154 nm).

### 2.4 Antimicrobial Activity Test

All experimental material was previously autoclaved at 121 °C for 15 min, and all procedures were performed under aseptic conditions.^28^ The protocols used were adapted.^29,30^ Initially, the lyophilized disk of the standard bacterial strain inoculated into 5 mL of nutrient broth was incubated at 37 °C for 48 h in a bacteriological incubator. Three transfers were performed using a 1 μL platinum loop to other tubes containing 5 mL of nutrient broth, followed by incubation at 37 °C for 24 h. The bacterial cultures obtained were diluted in saline solution (0.9% NaCl) until reaching a final concentration of 10^5^ CFU mL^–1^ and were defined as Solution A. The initial concentration of all bacterial suspensions (including controls) was standardized at 10^5^ CFU mL^–1^. The solutions with catalysts T, 2AgT/V, and 10AgT/V were sonicated for 30 min. To each well of a sterile six-well microplate, 0.1 mL of the catalyst solution (equivalent to 0.01 g mL^–1^) and 3.9 mL of saline were added. The reaction was initiated by the addition of 1 mL of Solution A, totaling a final volume of 5 mL in each well. Using a vibrating platform, the microplates were kept under constant agitation at 350 rpm at 37 °C throughout the experimental period. The samples were exposed to three lighting conditions: no light (designated as dark), clear light (Manplex, 9 W, LED), and black light (Luatek, 36 W, fluorescent), with a standardized distance of 10 cm between the light source and the microplates. Aliquots of 5 μL were collected with a calibrated platinum loop at 0, 5, 10, and 15 min and immediately plated on nutrient agar plates, where the inoculum was absorbed for 15 min. All assays were conducted in triplicate, with plates incubated at 37 ± 2 °C for 24 h in a bacteriological incubator. Control assays, conducted under the same conditions but without the addition of catalysts, were also performed. Finally, colony-forming units (CFU) were quantified and the data were statistically analyzed to evaluate the antimicrobial efficacy of the T, 2AgT/V, and 10AgT/V catalysts tested.

### 2.5 Detection of Reactive Oxygen Species (ROS)

The protocol established, with adaptations, for the detection of ROS in bacterial samples was used.^31^ The experimental procedure described in Section 2.4 was repeated, with the only difference being the bacterial dilution, whose concentration was 1 × 10^3^ CFU mL^–1^. After the defined reaction times, the collected samples were washed with phosphate-buffered saline (PBS, 1 mol L^–1^) and centrifuged at 3000 rpm for 5 min. The supernatant was then discarded, and the bacterial pellet was incubated in the dark at 37 °C for 30 min with 1 mL of 2′,7′-dichlorofluorescein diacetate (H_2_DCF-DA) solution prepared in PBS. After the incubation time, the cells were centrifuged at 5000 rpm for 6 min to remove excess H_2_DCF-DA. The cell pellets were then resuspended in 1 mL of PBS and mixed with 200 μL of alkaline lysis buffer (1% SDS; 0.2 mol L^–1^ NaOH) and incubated for 10 min at 37 °C. At the end of the process, the mixture was centrifuged again at 5000 rpm for 6 min, and the final supernatant was collected for fluorescence reads under optical density (excitation at 488 nm and emission at 525 nm) using the Thermo Scientific Varioskan LUX equipment. The results were expressed as a percentage (%) of ROS, relative to the initial time (0 min).

### 2.6 Statistical Analysis

GraphPad Prism 9.0, test version for academics, was used to perform the statistical analyses. Normality was analyzed by Kolmogorov–Smirnov, while the Grubbs test excluded outliers. Each experiment was carried out in triplicate and the differences between the groups in relation to the study variables were verified by one-way analysis of variance (ANOVA) according to the number of variables analyzed in the trial. Tukey’s post-test was then applied. Variations with a *p*-value <0.05 were considered statistically significant. The data was shown graphically and represented as mean and standard deviation.

## 3 Results and Discussion

### 3.1 Characterization of the Catalysts

The FT-IR spectra of the T, 2AgT/V and 10AgT/V catalysts are shown in Figure 2. These catalysts show characteristic titanium (Ti) bands between 500 and 750 cm^–1^ which have been correlated to the oxygen–titanium bond (Ti–O–Ti). Therefore, the band observed at 739 cm^–1^ belonging to the T catalyst intensified and broadened in the AgT/V catalysts, indicating that the Ti–O–Ti stretching vibration became stronger. This suggests that the addition of Ag and tapioca promoted a more robust Ti–O–Ti network, likely due to structural rearrangement during the doping process.^32–34^ Related to the 3000 cm^–1^ region of the T catalysts is the stretching vibration of the OH group.^35,36^ The absorption band for the 2AgT/V and 10AgT/V catalysts at 3480 cm^–1^ correlated to the stretching of the free −OH group, which was related to the increase in the number of H bonds between TiO_2_ and the OH group. The identification of hydroxyl groups on the surface is an important factor in improving antimicrobial performance; these groups act as traps for photogenerated gaps, promoting the formation of hydroxyl radicals, which are agents in the oxidation of the bacterial cell wall.^34^ In addition, the band at 1626 cm^–1^ belonging to the 2AgT/V and 10AgT/V catalysts refers to C–O stretching,^36–38^ and can also be attributed to the stretching and bending vibration of the OH group.^38^ The band present for these catalysts at 1400 cm^–1^ was characteristic of C–C stretching.^39^ Samples 2AgT/V and 10AgT/V contain residual organic components from the tapioca used in green synthesis. These organic residues can act as coating agents that stabilize Ag nanoparticles, improving the interaction between the catalyst surface and bacterial membranes.

**Figure 2:**
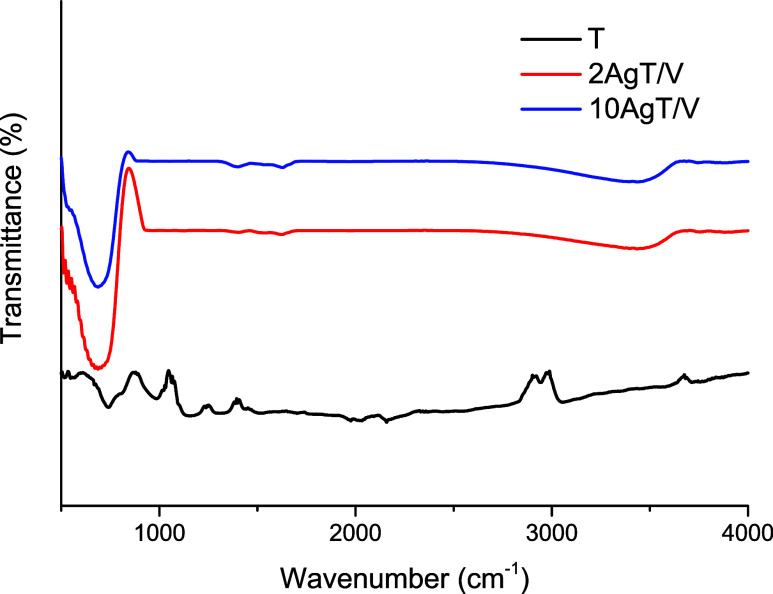
FT-IR spectra of the catalysts. FT-IR spectra of catalysts T, 2AgT/V, and 10AgT/V. Silver doping promoted changes in the structural vibrations of TiO_2_, evidencing greater interaction between Ag and TiO_2_ and an increase in −OH groups on the surface. The observed changes confirm the incorporation of silver and the modification of the surface structure after green synthesis.

There is a weak band at 1350 cm^–1^ for the AgT/V catalysts, which is characteristic of the Ag–TiO_2_ bond,^40^ confirming the deposition of silver on the support and highlighting the XRD results. These signals indicate that Ag is chemically bonded to the titania support. This interfacial bonding aids in the formation of a Schottky barrier, which effectively reduces electron–hole pair recombination, thus increasing the quantum efficiency of the photocatalytic process. It is estimated that Ag doping and the use of tapioca can improve the surface condition of the samples, generating more OH on the surface.^38^ Thanks to the addition of Ag to the 2AgT/V and 10AgT/V catalysts, there is a slight shift in the bands around 660–700 cm^–1^, correlated to the asymmetric vibration of Ti–Ag–O.^41^ It can be seen that the intensities of the bands of the 2AgT/V and 10AgT/V catalysts are lower compared to those of the T catalyst, thus indicating that the Ag particles bonded to the TiO_2_ after the green synthesis and successful surface modification.^13^

Figure 3 shows the diffractograms of the T, 2AgT/V and 10AgT/V catalysts. There are diffraction peaks outlined for catalyst T representing organized crystalline phases, containing two phases: anatase (JCPDS 21–1272), which was more abundant, and rutile (JCPDS 71–0650). The anatase phase had intense peaks at 25.4, 37.9, 48.1, 53.9, 55.2 and 62.8°. The rutile phase had peaks at 27.6, 29.8 and 44.4°.^11,42^ Catalyst T exhibited high crystallinity and a greater presence of anatase phases, which may interfere with its antimicrobial performance, suggesting that the intrinsic charge recombination rate of this catalyst remains a limiting factor for its effectiveness.

**Figure 3:**
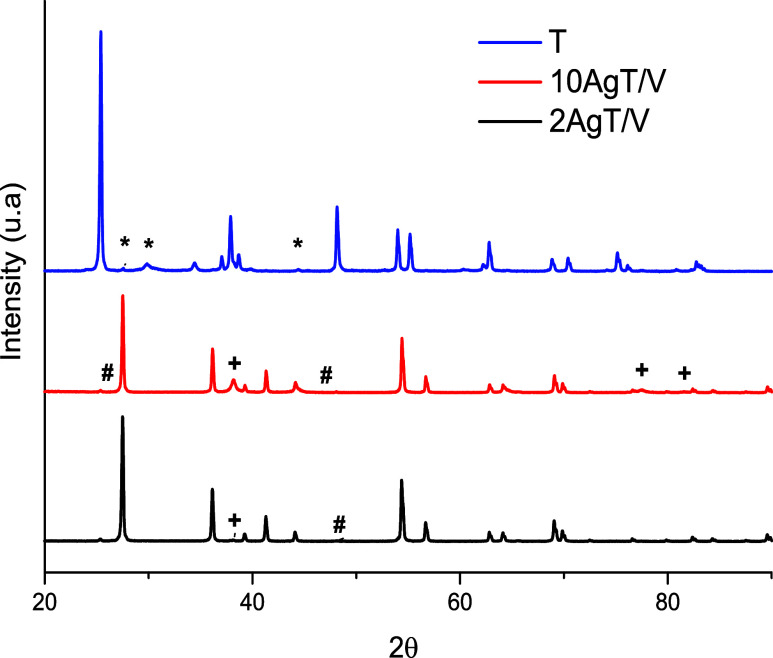
Diffractograms of catalysts the * identifies rutile while the other peaks of the T catalyst represent the anatase phase. The # identifies the anatase phase and the + stands for metallic Ag. The other peaks present in the AgT/V catalysts are the rutile phase.

The 2AgT/V and 10AgT/V catalysts have diffraction patterns characteristic of the cubic Ag phase (JCPDS 87–0717) with a peak at 38.2°. The identification of this reflection provides structural evidence of the reduction of Ag^+^ ions to Ag° during the green synthesis process. The starch components likely acted as reducing and stabilizing agents, ensuring the formation of the metallic phase without the need for toxic chemical reductants. Peak with low intensity in the tetragonal anatase phase at 48.1° (JCPDS 83–2243). The detection of Ag peaks in AgT/V catalysts confirms the diffusion and deposition of Ag atoms deposited on the TiO_2_ support.^43,44^ This structural arrangement is fundamental to the antimicrobial efficacy of the catalysts. The presence of metallic Ag nanoparticles on the TiO_2_ surface acts as a network for capturing photogenerated electrons, allowing holes to remain available for ROS generation. Thus, the XRD results can elucidate the performance of the 10AgT/V catalyst, where the higher Ag content provided more active sites for charge separation compared to catalyst T.

Research on polysaccharide-based sol–gel synthesis demonstrates distinct effects on the physicochemical properties of TiO_2_ compared to conventional methods. In general terms, a green sol–gel using polysaccharide tends to produce TiO_2_ with a more porous surface, functionalized with organic/oxygenated groups and a slightly reduced band gap, compared to conventional TiO_2_ sol–gel. Polysaccharide networks can generate TiO_2_ nanofibers or nanospheres well dispersed in a polymeric mesh.^45,46^

The tapioca offers a unique branched structure (amylopectin) that acts as a highly efficient three-dimensional template for Ag nanoparticle dispersion, and that acts as a template and stabilizer. The starch-mediated synthesis using cassava (tapioca) modifies the physicochemical surface of TiO_2_ by providing a matrix rich in hydroxyl groups, as evidenced by the intense band at 3480 cm^–1^ in the FT-IR spectrum. Unlike other polysaccharides, the high proportion of amylopectin in tapioca may facilitates the coordination of Ag^+^ ions at multiple binding sites, resulting in a more homogeneous distribution of metallic silver nanoparticles (Ag^0^). Ag^0^ was identified primarily in the AgT/V catalysts; however, smaller fractions of silver oxide are possible. The presence of species on the catalyst surface cannot be completely ruled out, given the oxidative atmosphere of calcination. These species, even if below the detection limit by XRD, can form beneficial heterojunctions that enhance the synergistic antimicrobial effect between Ag and TiO_2_.^47–49^

Presenting residual oxygenated carbon on the surface after moderate calcination, promoting higher density of hydroxyl/carboxyl groups and modified acidity/surface charge, effects generally more pronounced than in routes with neutral synthetic polymers.^47–49^ Tapioca promotes the formation of a single anatase phase in TiO_2_ nanoparticles, while other organic templates, such as ginger extract, facilitate the development of an anatase–rutile double phase.^50^ The use of activated starch templates creates polyhydroxylated nucleation sites that allow controlled growth of nanoparticles.^51^ Polysaccharide networks allow the controlled synthesis of TiO_2_ nanospheres with diameters ranging from 50 to 500 nm, where synthesis parameters, including polysaccharide concentration, temperature, and time, influence the particle characteristics.^46^ Green synthesis, which uses plant extracts as bioreducing agents and coating agents, enhances functional properties, producing TiO_2_ nanoparticles with superior photocatalytic activity, antibacterial effects, and antioxidant potential compared to chemically synthesized variants.^52^

### 3.2 Antimicrobial Activity Test

Figures 4(A–I) and 5(A–I) show the effects of visible light sources (called clear light), no light (called dark) and black light for: controls (A, D, and G) and the catalysts 2AgT/V (B, E, and H) and 10AgT/V (C, F, and I) on the inactivation of *E. coli* and *S. aureus*, respectively.

**Figure 4:**
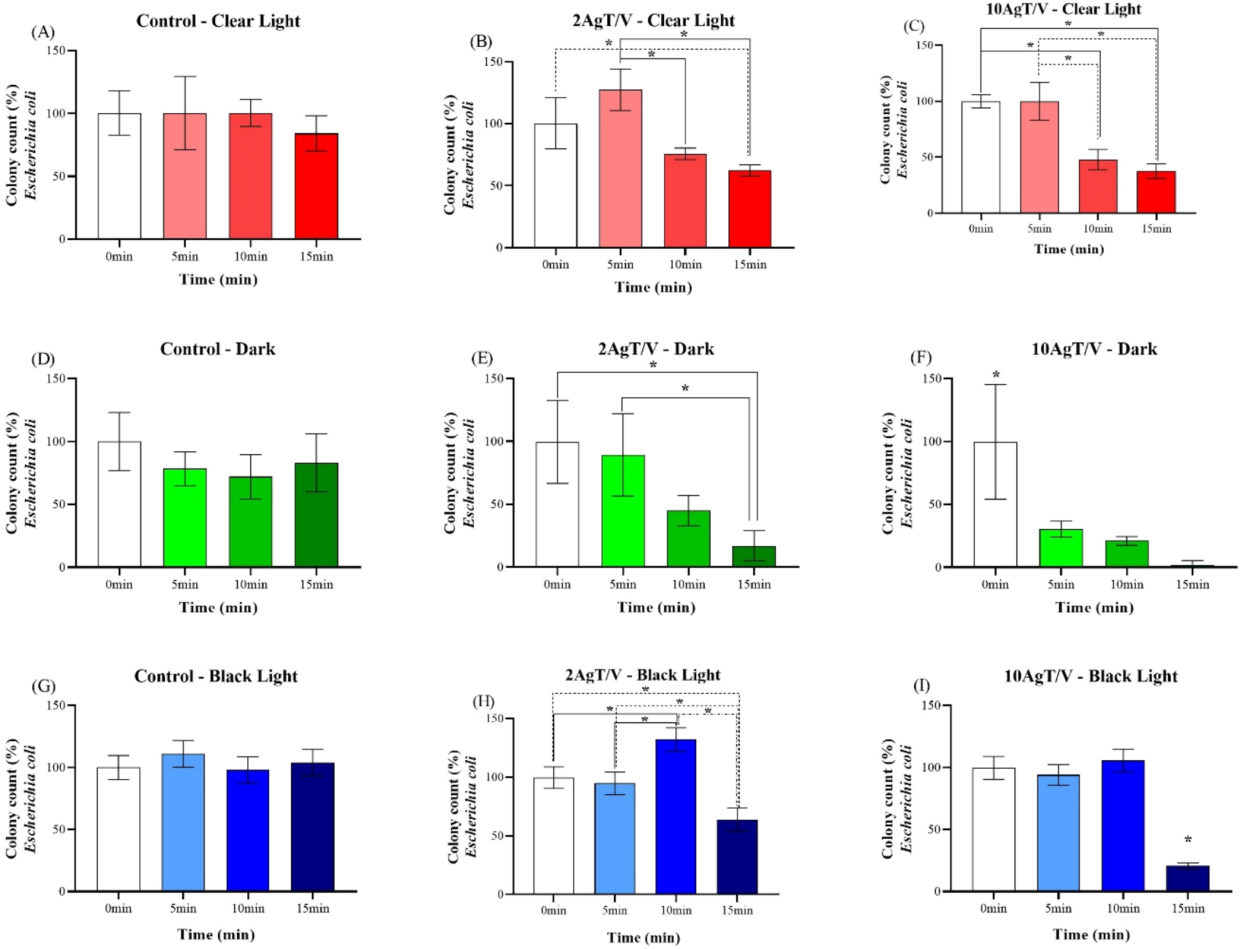
Antibacterial activity test (*E. coli*) of 2AgT/V and 10AgT/V catalysts under clear light (A–C), dark (D–F) and black light (G–I). Evaluation of antibacterial activity against *E. coli*, expressed as a percentage of the mean ± standard deviation, in colony count as a function of exposure time (0, 5, 10, and 15 min), with the initial time (0 min) normalized to 100%. Experimental conditions include, the control (A, D, G), the 2AgT/V catalysts (B, E, H) and 10AgT/V catalysts (C, F, I) which were tested under clear light (A–C), dark (D–F), and black light (G–I). Results are expressed as the mean of three independent replicates (*n* = 3), and error bars represent the standard deviation. Asterisks (*) indicate *p* < 0.05 (one-way ANOVA, Tukey’s posthoc test).

**Figure 5:**
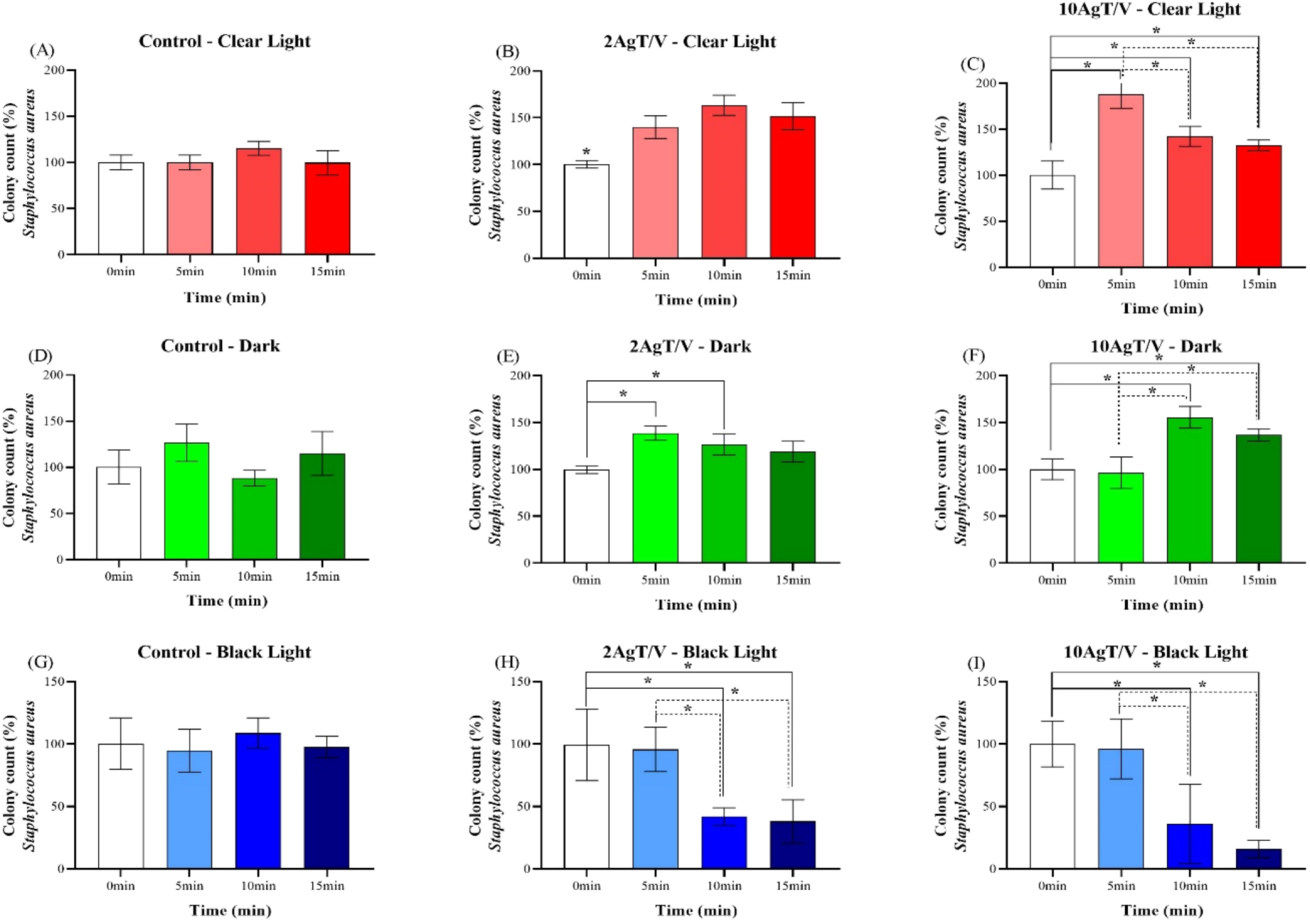
Antibacterial activity test (*S. aureus*) of 2AgT/V and 10AgT/V catalysts under clear light (A–C), dark (D–F) and black light (G–I). Evaluation of antibacterial activity against *S. aureus*, expressed as a percentage of the mean ± standard deviation, in colony count as a function of exposure time (0, 5, 10, and 15 min), with the initial time (0 min) normalized to 100%. Experimental conditions include, the control (A, D, G), the 2AgT/V catalysts (B, E, H) and 10AgT/V catalysts (C, F, I) which were tested under clear light (A–C), dark (D–F), and black light (G–I). Results are expressed as the mean of three independent replicates (*n* = 3), and error bars represent the standard deviation. Asterisks (*) indicate *p* < 0.05 (one-way ANOVA, Tukey’s posthoc test).

The 2AgT/V and 10AgT/V catalysts (Figure 4B,C), under white light, showed a reduction in bacterial growth against *E. coli*. After 15 min of exposure, the observed reductions were 38% and 63% for the 2AgT/V and 10AgT/V catalysts, respectively. In the dark test, the 2AgT/V catalyst (Figure 4E) exhibited antibacterial activity after 15 min, with an 83% reduction in cell viability. The 10AgT/V (Figure 4F) catalyst demonstrated superior performance, achieving 70% inhibition of *E. coli* in just 5 min and reaching 98% inactivation after 15 min in the dark. Bacterial inhibition in the dark indicated that Ag nanoparticles are primarily responsible for the toxicity. The increased efficacy of 10AgT/V (98% inactivation after 15 min) compared to 2AgT/V suggested a dose-dependent mechanism. This action may be attributed to the adhesion of Ag nanoparticles to the cell membrane, leading to structural damage and leakage of intracellular contents. Bacterial inhibition in the dark indicated that Ag nanoparticles are primarily responsible for the toxicity. The increased efficacy of the 10AgT/V catalyst (98% inactivation after 15 min) compared to 2AgT/V suggests a dose-dependent mechanism. This microbial inhibition may be related to the Ag nanoparticles released by the AgT/V catalysts; therefore, the amount of this metal in the catalyst affects its antimicrobial action. By introducing more Ag into the TiO_2_ matrix, the bactericidal action increases and acts through the adhesion of these nanoparticles to the surface of the cell membrane and wall, where they accumulate, causing leakage from intracellular organelles and leading to cell death.^53–55^ Ag nanoparticles can also enter the interior of the bacterial cell, causing damage to intracellular organelles, lipids, DNA and proteins. Ag can also incite cell toxicity and oxidative stress thanks to the generation of ROS.^56^ Thus, the bacterial inhibition of these catalysts may come from the oxidative stress generated by the high concentration of Ag nanoparticles or from the presence of Ag ions on their surface, in addition to the action of ROS.^57^ These AgT/V catalysts are effective against *E. coli* without photoactivation, due to the release of Ag ions from the TiO_2_ matrix. This result may be due to the fact that Ag has a powerful contact antibacterial action, and the amount of this metal increases its action. In addition, Ag could cause a decrease in soluble protein expression by suppressing nucleic acid synthesis, inhibiting *S. aureus*.^58^

It was observed that the 2AgT/V catalyst (Figure 4H), under black light irradiation for 10 min, showed a 32% bacterial growth. This behavior suggests that UV radiation, at an initial sublethal dose, may have favored photorepair and cell regeneration mechanisms in bacteria.^59,60^ However, with the prolongation of the time to 15 min, the synergistic action between Ag^+^ ions and ROS generated by TiO_2_ overcame the biological defense mechanism, promoting an inhibition of 36%. The 2AgT/V and 10AgT/V catalysts (Figure 5H,I, respectively) showed a decrease in bacterial growth of 62% and 84% after 15 min, respectively, against *S. aureus*. Meanwhile, the 10AgT/V catalyst (Figure 4I) against *E. coli*, after 15 min, showed an 85% reduction in bacterial growth. This superior performance is attributed to the photoactivation of the catalysts by UV radiation, thus causing the generation of ROS and intense photocatalytic activity, as well as the synergistic action of Ag.^61^

Another point that may have contributed to the efficiency of AgT/V catalysts is the tapioca residue on their surface. This was identified by the C–C and C–O stretching bands in FT-IR, the synthesis of tapioca-mediated AgT/V catalysts showed residual starch chains remaining anchored to their surface. These residual chains may improve catalyst performance through favorable biochemical interactions with the cell wall, overcoming the electrostatic repulsion often found in pure metal oxides. The organic matrix of tapioca may filter and mediate bacterial contact with Ag particles and ROS diffusion. This occurs due to increased roughness and anchoring areas, which potentially increase adhesion. It is important to consider that bacterial adhesion depends on topography/rigidity, surface roughness, wettability (hydrophilic vs hydrophobic), hydration layer, and the presence of an organic polymeric matrix around the inorganic particles. Superhydrophilic surfaces form a water layer that favors release by ROS/Ag^+^, while superhydrophobic surfaces can also impede adhesion due to the presence of an air layer. Well-dispersed particles in a starch matrix increase surface roughness.^62–64^ Studies with modified cassava starch adhesives and Ag particles show that the polymeric phase controls wettability, roughness, and cohesion, while Ag confers antifungal/antibacterial activity. Therefore, tapioca residues on the surface of AgT/V catalysts can indeed influence both bacterial adhesion and the effectiveness of ROS/Ag^+^, acting as a polymeric matrix that controls topography, wettability, and diffusion path. The key is the thickness/distribution of the starch layer and the exposed fraction of AgT/V catalyst.^64^

Contrary to expectations, the 2AgT/V and 10AgT/V catalysts showed increased bacterial growth under clear light and in the absence of light against *S. aureus*. Under clear light, an increase of 52% (Figure 5B) and 33% (Figure 5C) was observed, while in the dark the increases were 19% (Figure 5E) and 37% (Figure 5F). This Gram-positive bacterium has a cell wall rich in peptidoglycan, and its hydrophobic nature may confer variable resistance to oxidative stress. Additionally, this limitation in antibacterial activity may be attributed to the agglomeration of the AgT/V catalyst particles, reducing the surface area of contact and photocatalytic efficiency. Since the size and dispersion of Ag nanoparticles on TiO_2_ are crucial for bactericidal action, with Ag agglomerates smaller than 5 nm showing superior efficacy, the presence of larger aggregates may have compromised the biological performance of the tested AgT/V catalysts.^62^ Another possibility is that the AgT/V catalysts could cause a mild lesion in the *S. aureus* membrane, altering the membrane’s permeability but not causing its decomposition. The bacterial membrane has negative charges on its surface and the Ag nanoparticles released by the AgT/V catalyst come into contact with this surface, thus modifying its charge and hydrophobicity, affecting cell permeability. However, these modifications do not harm whole cells or induce leakage of macromolecules.^58^

The results presented showed that AgT/V catalysts possess antibacterial activity, even in the absence of light (against *E. coli*), indicating that Ag nanoparticles were responsible for the antimicrobial effect in the dark. The greater antibacterial activity of these catalysts under black light is due to the synergistic action between the photocatalytic reaction, originating from TiO_2_, and the Ag nanoparticles. The reduction in performance against *S. aureus* highlights that the response to AgT/V catalysts was dependent on the structural characteristics of the microorganisms, such as the composition and thickness of the peptidoglycan cell wall in Gram-positive bacteria. The versatility of AgT/V catalysts in acting under different light conditions and against different bacterial species significantly expands their application possibilities. Therefore, these materials are promising candidates for the development of functional antibacterial coatings, self-cleaning surfaces, and other applications.^65^

### 3.3 Detection of Reactive Oxygen Species

Figures 6(A, B) and 7(A,B) show the intracellular ROS generation values of *E. coli* and *S. aureus*, respectively, for the 2AgT/V (A) and 10AgT/V (B) catalysts, under black light. Intracellular ROS levels were monitored after standardized cell lysis, ensuring that fluorescence signals reflected oxidative stress within the bacteria. By maintaining a constant initial inoculum of 1 × 10^3^ CFU mL^–1^ and expressing the data as a percentage increase relative to the control *t* = 0, we effectively isolated the photocatalytic contribution of AgT/V catalysts to ROS overproduction. The use of an alkaline lysis buffer (1% SDS; 0.2 mol L-1 NaOH) ensured that the oxidized H2DCF-DA probe was fully released from the intracellular environment, allowing for a more precise quantification of the actual oxidative stress experienced by the bacteria, compared to simplified extracellular measurements.

**Figure 6:**
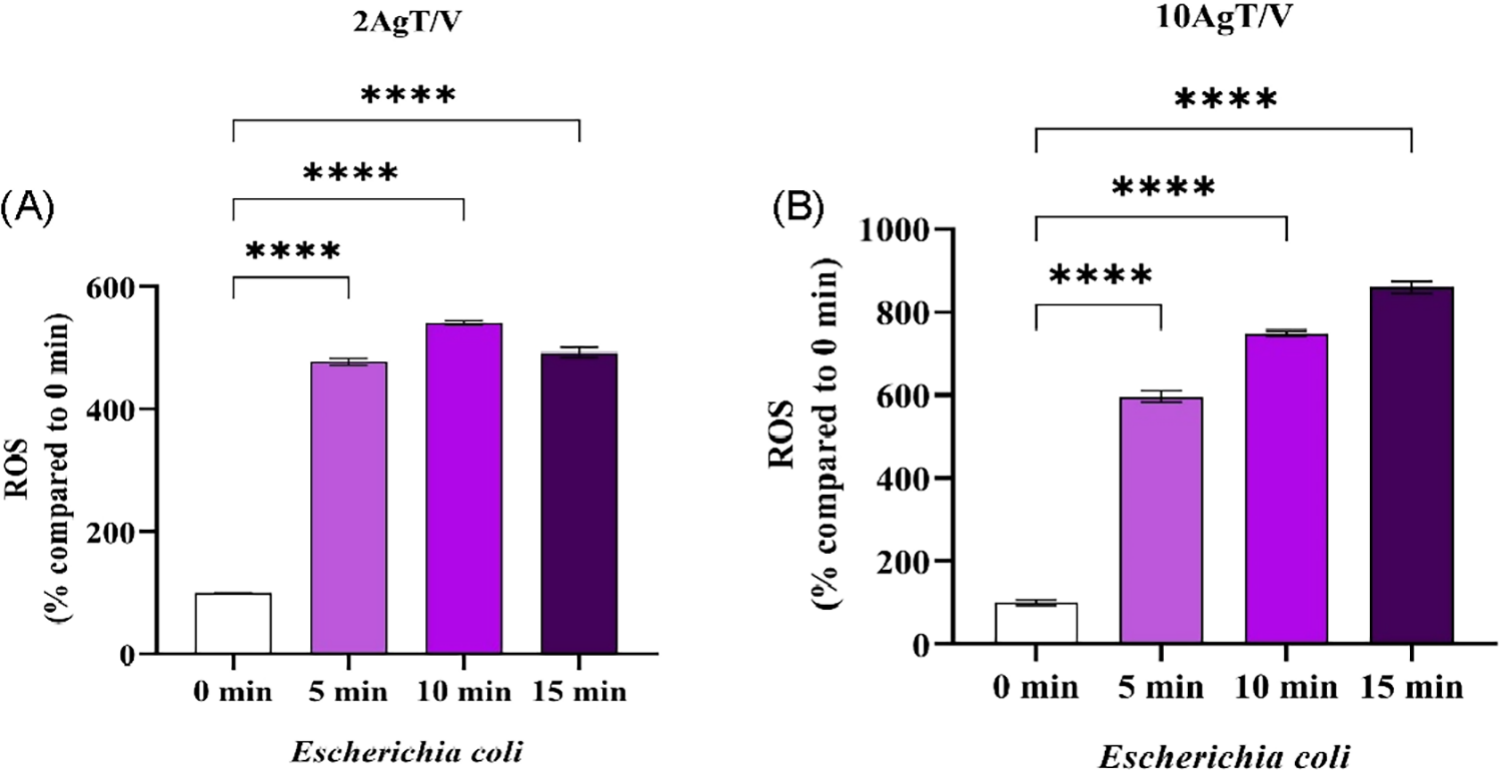
*E. coli* intracellular ROS generation values from the H_2_DCF-DA ROS detection assay for the 2AgT/V (A) and 10AgT/V (B) catalysts. Intracellular ROS generation values of *E. coli* for catalysts (A) 2AgT/V and (B) 10AgT/V. Results are expressed as the mean of three independent replicates (*n* = 3), and error bars represent the standard deviation. Asterisks indicate *p < 0.05; **p < 0.01; ***p < 0.001 and ****p < 0.0001 (one-way ANOVA, with Dunnett’s posthoc test).

**Figure 7:**
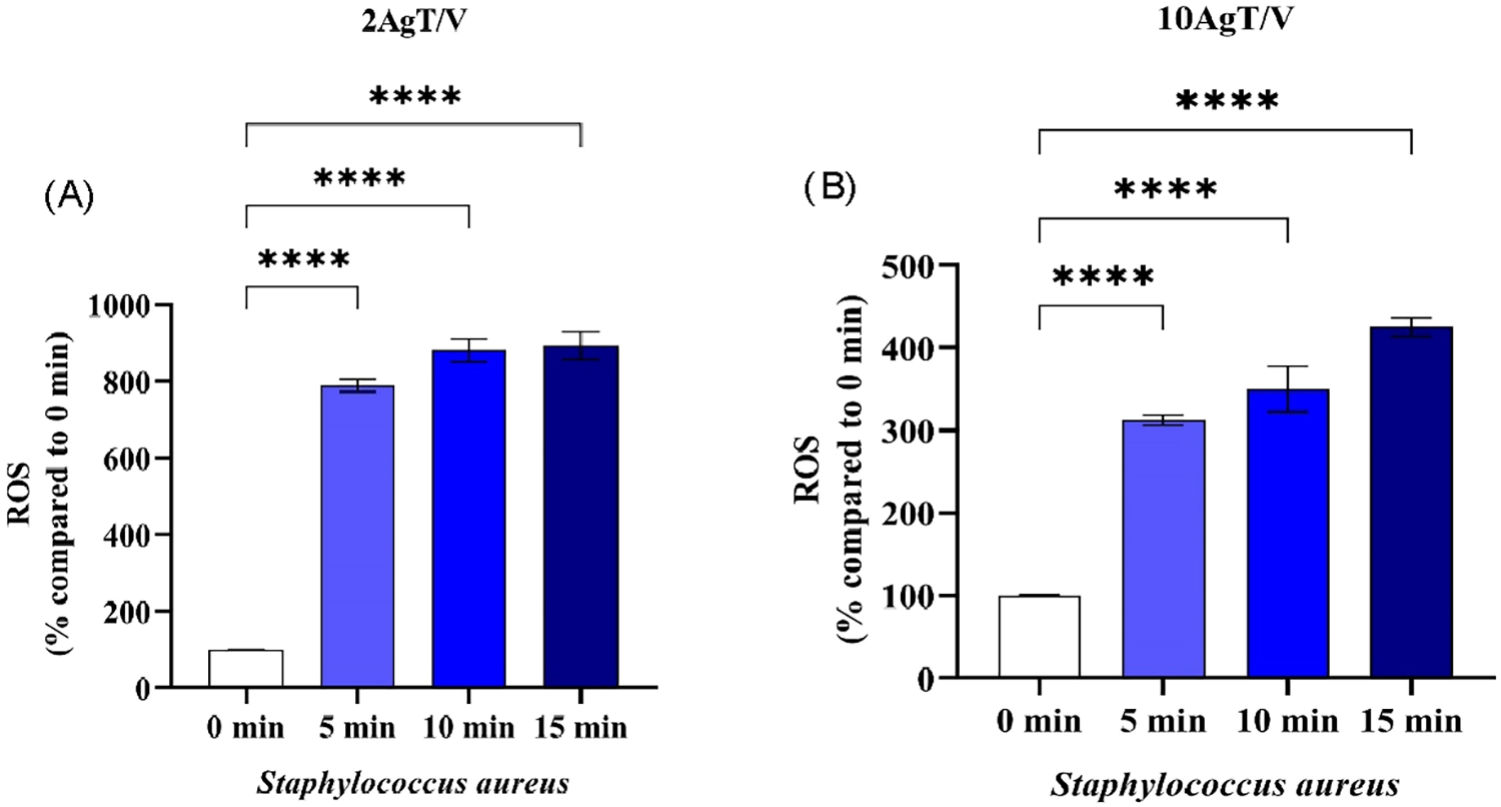
*S. aureus* intracellular ROS generation values from the H_2_DCF-DA ROS detection assay for the 2AgT/V (A) and 10AgT/V (B) catalysts. Intracellular ROS generation values of *S. aureus* for catalysts (A) 2AgT/V and (B) 10AgT/V. Results are expressed as the mean of three independent replicates (*n* = 3), and error bars represent the standard deviation. Asterisks indicate *p < 0.05; **p < 0.01; ***p < 0.001 and ****p < 0.0001 (one-way ANOVA, with Dunnett’s posthoc test).

The level of ROS was determined using exposure to the 2AgT/V and 10AgT/V catalysts, observing an increase in these levels from the 5 min time point for *E. coli* (Figure 6) and *S. aureus* (Figure 7). These results corroborate the data presented in Figure 4H,I, as well as 5H and I, i.e., the ROS were generated from the photocatalytic effects. These species accumulate and overload the bacteria’s antioxidant defense mechanisms, resulting in membrane damage and cell death.^66^ A greater production of ROS was observed for 10AgT/V in the death of *E. coli* (Figure 6B), while 2AgT/V stood out for *S. aureus* (Figure 7A). This response may be related to the specific interaction between Ag particles and peptidoglycan layers. Therefore, the thinner cell wall and the presence of porins in the Gram-negative bacterium *E. coli* may allow for a faster diffusion of generated ROS and Ag^+^ ions released by the 10AgT/V catalyst. However, the thicker cell wall of the Gram-positive bacterium *S. aureus* may require a more homogeneous distribution of Ag nanoparticles, achieved in the 2AgT/V sample, to effectively interact with cell wall sites and induce the observed intracellular redox imbalance. Thus, the diversity of microorganisms, such as the complexity and thickness of the cell wall, which interferes with the response to catalysts.^65^

AgT/V catalysts have a bactericidal mechanism of action that depends on oxidative stress and may be related to a chain of reactions. ROS are generated during bacterial aerobic metabolism. The constant generation and detoxification of cellular ROS act to control the fine and balanced redox state in normal bacterial cells. However, an imbalance between ROS production and detoxification can arise due to the overproduction of intracellular ROS (Figure 8). Initially, they are attracted to the bacterial surface, disrupting the cell wall and membrane, modifying their permeability, inciting toxicity and oxidative stress due to ROS and free radicals, and ending up modulating signal transduction pathways.^67^ The photoexcitation of TiO_2_ by UV light generates e^–^/h^+^ pairs found in the conduction and valence bands, respectively. Therefore, the e^–^ transferred from Ag/TiO_2_ to the bacterial intracellular part can affect the e^–^ transport chain, thus causing the overgeneration of intracellular ROS. These produced e^–^/h^+^ pairs collide with compounds present in the cellular environment, such as water and oxygen (O_2_), generating ^•^OH and O_2_^–•^ radicals. The gaps segregate H_2_O into OH^–^ and H^+^. The dissolved O_2_ molecules change into O_2_^–•^ which reacts with H^+^ to generate HO_2_^•^. These radicals collide with e^–^ producing hydrogen peroxide anions (HO_2_^–^). The anions, in turn, react with H^+^ ions to generate H_2_O_2_.^68^ At the same time, the electrons in TiO_2_’s conduction band are captured by the Ag nanoparticles and transported to O_2_^–•^, generating superoxide radicals. This metal also helps to generate ^•^OH radicals produced by the reaction of H_2_O with the photoproduced h^+^, located in the valence band of TiO_2_. The photogenerated ROS can cross the cell membrane and damage macromolecules such as proteins, DNA and lipids, thus altering biological activity, accelerating mutagenesis and bacterial death. Metallic Ag present in AgT/V catalysts acts as an electron trap, reduces recombination, and creates localized regions of high ROS generation at the catalyst-bacteria interface, which explains the accelerated death kinetics (15 min) observed compared to the T catalyst. In addition, the e^–^ donated Ag/TiO_2_ induces the production of extracellular O_2_^–•^ and H_2_O_2_, which can increase the endogenous generation of ROS.^69,70^

**Figure 8:**
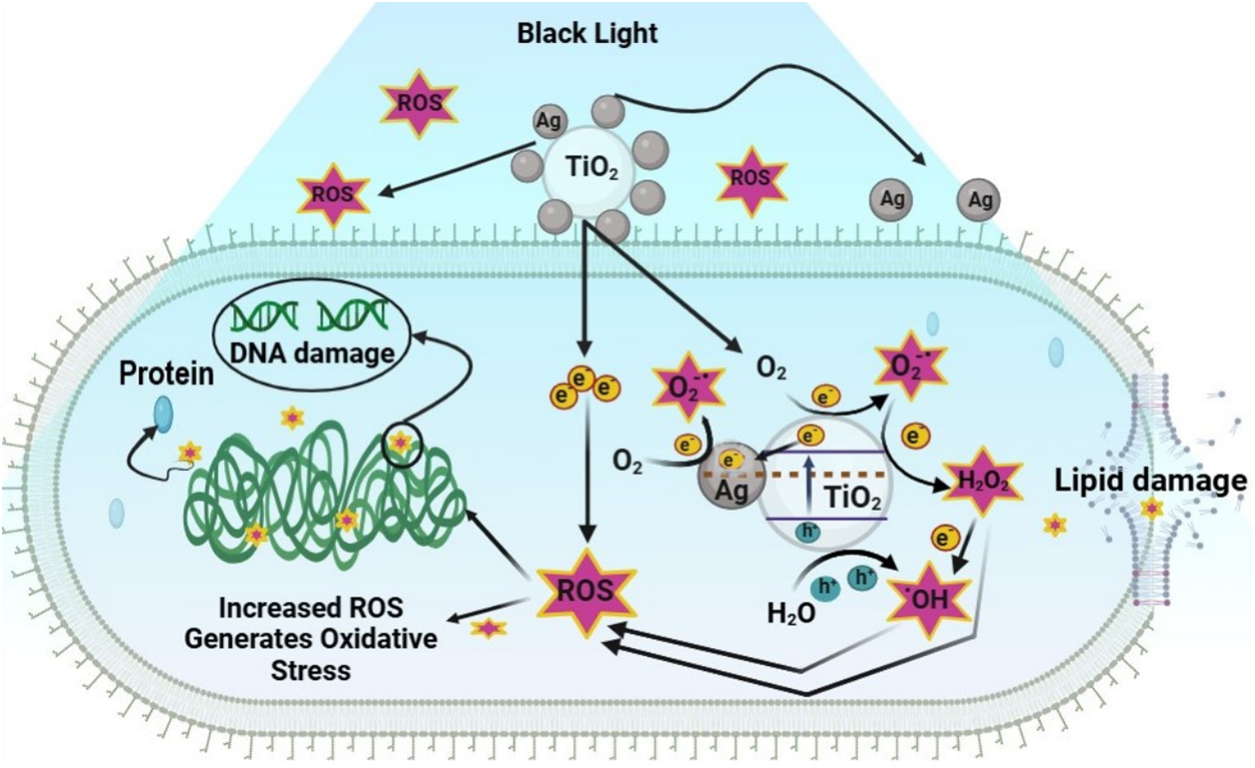
Illustration of the mechanism of action of AgT/V catalysts The AgT/V catalyst, when exposed to light, generates electrons (e^–^) and gaps (h^+^) which migrate to the surface of the TiO_2_. Inside the bacterial cell, the e^–^/h^+^ pairs migrate to the surface of the TiO_2_, where they react with water and molecular oxygen. The silver (Ag) present in the catalyst acts as an efficient e-acceptor, intensifying the production of ROS, such as superoxide radicals (O_2_^–•^), hydroxyl radicals (HO^•^), hydrogen peroxide (H_2_O_2_) and singlet oxygen (^1^O_2_). These highly reactive molecules penetrate the bacteria, damage the DNA and the cell membrane, overloading the antioxidant defense mechanisms and culminating in cell death.

### 3.4 Cytotoxicity and Environmental Implications of the Ag/TiO_2_ Catalyst

The potential application of Ag/TiO_2_ catalysts requires a comprehensive understanding of their safety profile. Although our results demonstrate effective antibacterial action, the prolonged use of Ag-based nanomaterials raises concerns regarding Ag leaching and its subsequent biological impact. In the AgT/V system, the cassava starch matrix plays a dual role: it acts as a stabilizing agent that improves the anchoring of silver on the TiO_2_ surface, potentially reducing uncontrolled leaching, but also determines the surface chemistry that governs the interaction with biological membranes. Ag^+^ ions from Ag/TiO_2_ catalysts are released,^71–73^ however, the extent of release varies greatly with the synthesis method, matrix, and reaction medium. In many well-anchored systems, release is low. For Ag/TiO_2_ films synthesized with starch, the migration of Ag to food simulants (water, acetic acid, and ethanol) resulted in a maximum release measurement of 0.141 mg/dm^2^ in acetic acid. This value was below the regulatory limits of MERCOSUR and the European Commission (8 and 10 mg/dm^2^, respectively).^74^ This indicates that release occurs, but the starch matrix acts as a reducing and stabilizing agent, decreasing the mobility and dissolution of Ag.^75^ Although green synthesis mediated by cassava starch has proven effective in stabilizing Ag nanoparticles on a TiO_2_ matrix, the dynamics of Ag^+^ ion release from these materials have not yet been fully explored. This gap represents an opportunity for future research aimed at evaluating the stability of the catalyst and its application potential.

The environmental implications are complex, since Ag nanoparticles undergo dissolution and oxidation processes, with Ag_2_S as the thermodynamically stable end product, in addition to exhibiting colloidal transformations that can facilitate the transport of other substances.^76^ In eco-nanocomposites chitosan/corn starch/SiO_2_@Ag obtained by sol–gel, Ag is released, in a solution formulated to replicate the inorganic elements present in blood plasma, at levels of 1.3–2.5 mol %, maintaining antibacterial activity and bioactivity (hydroxyapatite formation).^77^ Life cycle assessment studies indicate that the synthesis of silver nanoparticles stabilized with corn starch has negligible environmental impacts, with primary concerns arising from the production of metallic Ag and electricity consumption, rather than organic stabilizers. Stabilized Ag nanoparticles, synthesized using glucose and corn starch in a microwave-assisted reaction system, show that Ag production is the factor that contributes most to the environmental impact. It was also pointed out that microwave synthesis contributed to energy impacts. The starch and glucose used have a smaller contribution to the impact, being significant only in air pollutant emissions.^47^

Corn starch films with Ag particles show that the synthesis route strongly alters biocompatibility. Syntheses using corn starch as a stabilizer for Ag particles and deposition on TiO_2_ produce solution-stable catalysts after 320 days of aging, suggesting good physical retention of the nanoparticles; however, there was no detailed quantification of Ag leaching into water during photocatalytic cycles.^48,49^ The use of corn starch in the sol–gel synthesis of Ag/TiO_2_ improves the environmental profile of the process, but the released silver remains the main factor in cytotoxicity and ecotoxicity.

Based on studies on Ag/TiO_2_ supported on biomolecules, such as those immobilized on expanded clay, which maintained antibacterial activity after 4 cycles, showing a reduction efficiency greater than 4 log against *Vibrio parahemolyticus* and *V. harveyi*. The antibacterial efficiency decreased to 2.5–3.2 log in the seventh cycle.^78^ The Ag–Ag_2_S/TiO_2_ biocomposite supported on cellulose maintained an inactivation of *E. coli* from 97% to 87% after 4 cycles.^79^ Ag-TiO_2_ supported on *Allium cepa* (onion) peel extract decreased from 98% to approximately 72% efficiency after 4 cycles, associated with agglomeration and slight loss of Ag/Ti.^80^ Regarding shelf life, Ag-TiO_2_/ZIF-8 maintained effective antimicrobial activity for more than 5 months of storage.^81^ The cellulose/chitosan–Ag/TiO_2_ packaging preserved photocatalytic properties after 4 months. This suggests that if the green Ag/TiO_2_ catalysts are properly dried and protected from intense light and harsh environments, they can maintain activity for months. Thus, it is estimated that the material maintains its high antibacterial efficiency for at least 4 to 6 cycles of use, provided it undergoes washing protocols to remove cellular debris that may block the active sites, with a subsequent gradual decline.^82^ This topic will be investigated in future research.

AgT/V catalysts have demonstrated high efficiency in short-term bacterial inactivation, but future research is essential to validate their technical feasibility and safety on an industrial scale. For long-term applications, the aim is to quantify Ag leaching in multiple reuse cycles, ensuring material stability and maintaining its photocatalytic performance. Furthermore, the goal is to explore Ag anchoring in the TiO_2_ matrix to minimize metal losses during the decontamination process. Additionally, cytotoxicity assays in human cells and ecotoxicity assays in aquatic organisms are intended to ensure that the use of these materials is safe for both public health and the environment.

## 4 Conclusion

AgT/V catalysts were successfully synthesized via a modified sol–gel using tapioca as a reducing agent and sustainable stabilizer. The methodology is simple, sustainable, fast, ecological, efficient, and reproducible. This can facilitate large-scale production and reduce production costs due to the use of tapioca, thus favoring the potential commercial application of these catalysts. XRD and FT-IR characterizations confirmed the effective anchoring of Ag^0^ in the TiO_2_ matrix, the presence of hydroxyl groups on the surface, crucial for photocatalytic efficiency, and the preservation of the anatase–rutile mixed crystal structure. Antimicrobial assays demonstrated that the incorporation of Ag overcomes the limitations of pure TiO_2_. The AgT/V catalysts showed great potential for antibacterial applications, exhibiting rapid activity, up to 10 min, against bacteria. Specifically, the 10AgT/V catalyst achieved a 98% reduction in *E. coli* growth in 15 min in the dark and an 85% reduction under black light irradiation. Against *S. aureus*, the same catalyst showed an 84% reduction in bacterial growth after 15 min under black light. This rapid kinetic response suggests a synergistic effect between the action of Ag and the generation of ROS, indicating that the green synthesis process produces high-performance materials. AgT/V catalysts represent a promising alternative for the rapid decontamination of surfaces and water purification, offering a robust platform for future applications in the health, food, and environmental sectors by utilizing sustainable chemical compounds.

## References

[1] Pathak N., Caleb O. J., Rauh C., Mahajan P. V. (2019). Efficacy of Photocatalysis
and Photolysis Systems for the Removal of Ethylene under Different
Storage Conditions. Postharvest Biol. Technol..

[2] Ahmad I., Akhtar M. S., Ahmed E., Ahmad M. (2020). Highly Efficient Visible
Light Driven Photocatalytic Activity of Graphene and CNTs Based Mg
Doped ZnO Photocatalysts: A Comparative Study. Sep. Purif. Technol..

[3] Hlavsa M. C., Cikesh B. L., Roberts V. A., Kahler A. M., Vigar M., Hilborn E. D., Wade T. J., Roellig D. M., Murphy J. L., Xiao L., Yates K. M., Kunz J. M., Arduino M. J., Reddy S. C., Fullerton K. E., Cooley L. A., Beach M. J., Hill V. R., Yoder J. S. (2018). Outbreaks
Associated with Treated
Recreational Water—United States, 2000–2014. Am. J. Transplant..

[4] Pérez-Rodríguez F., Posada-Izquierdo G. D., Valero A., García-Gimeno R. M., Zurera G. (2013). Modelling Survival Kinetics of *Staphylococcus
aureus* and *Escherichia coli* O157:H7 on Stainless Steel Surfaces Soiled with Different Substrates
under Static Conditions of Temperature and Relative Humidity. Food Microbiol..

[5] Nag R., Monahan C., Whyte P., Markey B. K., O’Flaherty V., Bolton D., Fenton O., Richards K. G., Cummins E. (2021). Risk Assessment
of *Escherichia coli* in Bioaerosols
Generated Following Land Application of Farmyard Slurry. Sci. Total Environ..

[6] Ochiai T., Fujishima A. (2012). Photoelectrochemical
Properties of TiO_2_ Photocatalyst
and Its Applications for Environmental Purification. J. Photochem. Photobiol., C.

[7] Zacarías S. M., Satuf M. L., Vaccari M. C., Alfano O. M. (2015). Photocatalytic Inactivation
of Bacterial Spores Using TiO_2_ Films with Silver Deposits. Chem. Eng. J..

[8] Chandra S., Jagdale P., Medha I., Tiwari A., Bartoli M., Nino A., Olivito F. (2021). Biochar-Supported TiO_2_-Based Nanocomposites for the Photocatalytic Degradation of
Sulfamethoxazole
in Water—A Review. Toxics.

[9] Rajaram P., Jeice A. R., Jayakumar K. (2023). Review of
Green Synthesized TiO_2_ Nanoparticles for Diverse Applications. Surf. Interfaces.

[10] Rabhi S., Belkacemi H., Bououdina M., Kerrami A., Brahem L. A., Sakher E. (2019). Effect of Ag Doping
of TiO_2_ Nanoparticles
on Anatase-Rutile Phase Transformation and Excellent Photodegradation
of Amlodipine Besylate. Mater. Lett..

[11] Poudel M. B., Lohani P. C., Kim A. A. (2022). Synthesis
of Silver Nanoparticles
Decorated Tungsten Oxide Nanorods as High-Performance Supercapacitor
Electrode. Chem. Phys. Lett..

[12] Yin J., Lv L., Chu Y., Tan L. (2023). Highly Antibacterial Cu/Fe/N Co-Doped
TiO_2_ Nanopowder under Visible Light. Inorg. Chem. Commun..

[13] Poudel M. B., Kim A. A. (2023). Silver Nanoparticles Decorated TiO_2_ Nanoflakes
for Antibacterial Properties. Inorg. Chem. Commun..

[14] Padmavathi J., Mani M., Gokulakumar B., Ramesh A., Anantharaj A., Kaviyarasu K. (2022). A Study on
the Antibacterial Activity of Silver Nanoparticles
Derived from Corchorus Aestuans Leaves and Their Characterization. Chem. Phys. Lett..

[15] Saleem A., Iqbal A., Younas U., Ashraf A., Al-Mijalli S. H., Ali F., Pervaiz M., Saeed Z., Nazir A., Iqbal M. (2024). Antimicrobial
Attributes and Enhanced Catalytic Potential of PVA Stabilized Ag-NiO_2_ Nanocomposite for Wastewater Treatment. Arab. J. Chem..

[16] Ong C. B., Ng L. Y., Mohammad A. W. (2018). A Review of ZnO
Nanoparticles as
Solar Photocatalysts: Synthesis, Mechanisms and Applications. Renewable Sustainable Energy Rev..

[17] Gomes M. A., Brandão-Silva A. C., Avila J. F. M., Alencar M. A. R. C., Rodrigues J. J., Macedo Z. S. (2018). Particle Size Effect on Structural
and Optical Properties of Y_2_O_3_:Nd^3+^ Nanoparticles Prepared by Coconut Water-Assisted Sol-Gel Route. J. Lumin..

[18] Parveen, K. ; Banse, V. ; Ledwani, L. In Green Synthesis of Nanoparticles: Their Advantages and Disadvantages, AIP Conference Proceedings; AIP, 2016 10.1063/1.4945168.

[19] Gheisari F., Kasaee S. R., Mohamadian P., Chelliapan S., Gholizadeh R., Zareshahrabadi Z., Solhjoo S. P., Vafa E., Mosleh-Shirazi S., Amani A. M., Kamyab H. (2024). Bromelain-Loaded Silver
Nanoparticles: Formulation, Characterization and Biological Activity. Inorg. Chem. Commun..

[20] U.S. Department of Agriculture . FoodData Central Search Results: Mixed Nuts, 2025 https://fdc.nal.usda.gov/fdc-app.html#/food-search?query=&type=Foundation. (accessed October 20, 2025).

[21] Rengga W. D. P., Yufitasari A., Adi W. (2017). Synthesis of silver nanoparticles
from silver nitrate solution using green tea extract (*Camelia sinensis*) as bioreductor. J. Bahan Alam Terbarukan.

[22] Zakiyyah S. N., Irkham, Einaga Y., Gultom N. S., Fauzia R. P., Kadja G. T. M., Gaffar S., Ozsoz M., Hartati Y. W. (2024). Green Synthesis of Ceria Nanoparticles
from Cassava Tubers for Electrochemical Aptasensor Detection of SARS-CoV-2
on a Screen-Printed Carbon Electrode. ACS Appl.
Bio Mater..

[23] de
Almeida W. L., Rodembusch F. S., Ferreira N. S., de Sousa V. C. (2020). Eco-Friendly
and Cost-Effective Synthesis of ZnO Nanopowders by Tapioca-Assisted
Sol-Gel Route. Ceram. Int..

[24] de
O Primo J., de S Correa J., Horsth D. F. L., Das A., Zając M., Umek P., Wattiez R., Anaissi F. J., Onderwater R. C. A., Bittencourt C. (2022). Antiviral Properties against SARS-CoV-2
of Nanostructured ZnO Obtained by Green Combustion Synthesis and Coated
in Waterborne Acrylic Coatings. Nanomaterials.

[25] Khorrami G. H., Kompany A., Zak A. K. (2015). Structural and Optical Properties
of (K,Na)­NbO_3_ Nanoparticles Synthesized by a Modified Sol–Gel
Method Using Starch Media. Adv. Powder Technol..

[26] Tian X., Wen J., Wang S., Hu J., Li J., Peng H. (2016). Starch-Assisted
Synthesis and Optical Properties of ZnS Nanoparticles. Mater. Res. Bull..

[27] Ferreira N. S., Angélica R. S., Marques V. B., De Lima C. C. O., Silva M. S. (2016). Cassava-Starch-Assisted
Sol–Gel Synthesis of CeO_2_ Nanoparticles. Mater. Lett..

[28] Silva, N. ; Junqueira, V. C. A. ; Silveira, N. F. A. ; Taniwaki, M. H. ; Gomes, R. A. R. ; Okazaki, M. M. Manual de Métodos de Análise Microbiológica de Alimentos e Água., 5th ed.; Blucher: São Paulo, 2018.

[29] de
S Cordeiro A. C., Leite S. G. F., Dezotti M. (2004). Inativação
Por Oxidação Fotocatalítica de *Escherichia coli* e *Pseudomonas Sp.*. Quim. Nova.

[30] Tsai T., Chang H., Chang K., Liu Y., Tseng C. (2010). A Comparative
Study of the Bactericidal Effect of Photocatalytic Oxidation by TiO _2_ on Antibiotic-resistant and Antibiotic-sensitive Bacteria. J. Chem. Technol. Biotechnol..

[31] Liao S., Zhang Y., Pan X., Zhu F., Jiang C., Liu Q., Cheng Z., Dai G., Wu G., Wang L., Chen L. (2019). Antibacterial Activity and Mechanism
of Silver Nanoparticles against
Multidrug-Resistant *Pseudomonas aeruginosa*. Int. J. Nanomed..

[32] Alsharaeh E. H., Bora T., Soliman A., Ahmed F., Bharath G., Ghoniem M. G., Abu-Salah K. M., Dutta J. (2017). Sol-Gel-Assisted Microwave-Derived
Synthesis of Anatase Ag/TiO_2_/GO Nanohybrids toward Efficient
Visible Light Phenol Degradation. Catalysts.

[33] Safaei M., Taran M. (2017). Optimal Conditions for Producing Bactericidal Sodium Hyaluronate-TiO_2_ Bionanocomposite and Its Characterization. Int. J. Biol. Macromol..

[34] Noviagel I., Heryanto H., Putri S. E., Rauf I., Tahir D. (2024). Tapioca-Starch-Based
Bionanocomposites with Fructose and Titanium Dioxide for Food Packaging
and Fertilization Applications. Int. J. Biol.
Macromol..

[35] León A., Reuquen P., Garín C., Segura R., Vargas P., Zapata P., Orihuela P. (2017). FTIR and Raman Characterization of
TiO_2_ Nanoparticles Coated with Polyethylene Glycol as Carrier
for 2-Methoxyestradiol. Appl. Sci..

[36] Yang H., Dai K., Zhang J., Dawson G. (2022). Inorganic-Organic Hybrid Photocatalysts:
Syntheses, Mechanisms, and Applications. Chin.
J. Catal..

[37] Eleftheriadou N. M., Ofrydopoulou A., Papageorgiou M., Lambropoulou D. (2020). Development
of Novel Polymer Supported Nanocomposite GO/TiO_2_ Films,
Based on Poly­(L-Lactic Acid) for Photocatalytic Applications. Appl. Sci..

[38] Sathishkumar K., Sowmiya K., Pragasan L. A., Rajagopal R., Sathya R., Ragupathy S., Krishnakumar M., Reddy V. R. M. (2022). Enhanced Photocatalytic Degradation of Organic Pollutants
by Ag–TiO_2_ Loaded Cassava Stem Activated Carbon
under Sunlight Irradiation. Chemosphere.

[39] Ateş M., Kuz P. (2020). Starch-Based Bioplastic
Materials for Packaging Industry. J. Sustainable
Constr. Mater. Technol..

[40] Desiati R. D., Taspika M., Sugiarti E. (2019). Effect of
Calcination Temperature
on the Antibacterial Activity of TiO_2_/Ag Nanocomposite. Mater. Res. Express.

[41] Gogoi D., Namdeo A., Golder A. K., Peela N. R. (2020). Ag-Doped TiO_2_ Photocatalysts with Effective
Charge Transfer for Highly
Efficient Hydrogen Production through Water Splitting. Int. J. Hydrogen Energy.

[42] JCPDS . JCPDS—International Center for Diffraction Data, PCPDFWIN, 2013; Vol. 5, p 130.

[43] Sharma H., Singhal R., Kumar V. V. S., Asokan K. (2016). Structural, Optical
and Electronic Properties of Ag–TiO_2_ Nanocomposite
Thin Film. Appl. Phys. A: Mater. Sci. Process..

[44] Mosquera A. A., Endrino J. L., Albella J. M. (2014). Xanes Observations of the Inhibition
and Promotion of Anatase and Rutile Phases in Silver Containing Films. J. Anal. At. Spectrom..

[45] Flores-Gómez J., Mota-Macías S., Guerrero-Jiménez J. P., Romero-Arellano V. H., Morales-Rivera J. (2024). Sol–Gel Synthesis of TiO_2_ with Pectin
and Their Efficiency in Solar Cells Sensitized by Quantum Dots. Gels.

[46] Melendez C. L., Romero H. A. M., Carreño-Gallardo C., Mata G. M., Santiesteban R. P., Piñon T. P., Piñon D. P., Aguilar H. A. L., Macias M. E. E., Chacón-Nava J. G. (2024). Formation
of Olive-like TiO_2_ Nanospheres in a Polymeric Mesh by Sol-Gel
Method. Polymers.

[47] Bafana A., Kumar S. V., Temizel-Sekeryan S., Dahoumane S. A., Haselbach L., Jeffryes C. S. (2018). Evaluating Microwave-Synthesized
Silver Nanoparticles from Silver Nitrate with Life Cycle Assessment
Techniques. Sci. Total Environ..

[48] Strapasson G. B., Assis M., Backes C. W., Corrêa S. A., Longo E., Weibel D. E. (2021). Microwave Assisted
Synthesis of Silver
Nanoparticles and Its Application in Sustainable Photocatalytic Hydrogen
Evolution. Int. J. Hydrogen Energy.

[49] Ortega F., Minnaard J., Arce V. B., García M. A. (2025). Biodegradable
Starch-Based Nanocomposite Films with Laser-Synthesized Silver Nanoparticles:
A Materials Approach for Packaging. Int. J.
Biol. Macromol..

[50] Saikumari N., Sudhakhar K. S. (2023). Extensive
Function of Green Synthesized Titania Nanoparticles:
Photodegradation of Congo Red. TAPPI J..

[51] Saliu O. D., Mamo M., Ndungu P., Ramontja J. (2022). Starch Built TiO_2_ Nanoarchitecture with Mixed Anatase and Rutile Phase for
High Energy Density Supercapacitor Electrode. J. Energy Storage.

[52] Shakeel N., Piwoński I., Iqbal P., Kisielewska A. (2025). Green Synthesis
of Titanium Dioxide Nanoparticles: Physicochemical Characterization
and Applications: A Review. Int. J. Mol. Sci..

[53] Dong P., Yang F., Cheng X., Huang Z., Nie X., Xiao Y., Zhang X. (2019). Plasmon Enhanced Photocatalytic and
Antimicrobial Activities of Ag-TiO_2_ Nanocomposites under
Visible Light Irradiation Prepared by DBD Cold Plasma Treatment. Mater. Sci. Eng.: C.

[54] Ashkarran A. A., Aghigh S. M., Kavianipour M., Farahani N. J. (2011). Visible Light Photo-and
Bioactivity of Ag/TiO_2_ Nanocomposite with Various Silver
Contents. Curr. Appl. Phys..

[55] Chakhtouna H., Benzeid H., Zari N., el kacem Qaiss A., Bouhfid R. (2021). Recent Progress on Ag/TiO_2_ Photocatalysts:
Photocatalytic and Bactericidal Behaviors. Environ.
Sci. Pollut. Res..

[56] Tian J., Wong K. K. Y., Ho C., Lok C., Yu W., Che C., Chiu J., Tam P. K. H. (2007). Topical Delivery
of Silver Nanoparticles
Promotes Wound Healing. Chem. Med. Chem..

[57] Perkas N., Lipovsky A., Amirian G., Nitzan Y., Gedanken A. (2013). Biocidal Properties
of TiO_2_ Powder Modified with Ag Nanoparticles. J. Mater. Chem. B.

[58] Jiang X., Lv B., Wang Y., Shen Q., Wang X. (2017). Bactericidal Mechanisms
and Effector Targets of TiO_2_ and Ag-TiO_2_ against
Staphylococcus Aureus. J. Med. Microbiol..

[59] Chan Y. Y., Killick E. G. (1995). The Effect of Salinity, Light and Temperature in a
Disposal Environment on the Recovery of *E. coli* Following Exposure to Ultraviolet Radiation. Water Res..

[60] Tosa K., Hirata T. (1999). Photoreactivation of Enterohemorrhagic *Escherichia coli* Following UV Disinfection. Water Res..

[61] Dahl M., Liu Y., Yin Y. (2014). Composite Titanium
Dioxide Nanomaterials. Chem. Rev..

[62] Hajizadeh H., Peighambardoust S. J., Peighambardoust S. H., Peressini D. (2020). Physical,
Mechanical, and Antibacterial Characteristics of Bio-nanocomposite
Films Loaded with Ag-modified SiO_2_ and TiO_2_ Nanoparticles. J. Food Sci..

[63] Gui L., Lin J., Liu J., Zuo J., Wang Q., Jiang W., Feng T., Li S., Wang S., Liu Z. (2022). Difference
and Association of Antibacterial and Bacterial Anti-Adhesive Performances
between Smart Ag/AgCl/TiO_2_ Composite Surfaces with Switchable
Wettability. Chem. Eng. J..

[64] Jarensungnen C., Jetsrisuparb K., Phanthanawiboon S., Theerakulpisut S., Hiziroglu S., Knijnenburg J. T. N., Okhawilai M., Kasemsiri P. (2023). Development
of Eco-Friendly Antifungal and Antibacterial
Adhesive Derived from Modified Cassava Starch Waste/Polyvinyl Alcohol
Containing Green Synthesized Nano-Silver. Sci.
Rep..

[65] Zhang H., Chen G. (2009). Potent Antibacterial Activities of
Ag/TiO_2_ Nanocomposite
Powders Synthesized by a One-Pot Sol–Gel Method. Environ. Sci. Technol..

[66] Yong S.-S., Lee J.-I., Kang D.-H. (2023). TiO_2_-Based Photocatalyst
Generated Reactive Oxygen Species Cause Cell Membrane Disruption of *Staphylococcus Aureus* and *Escherichia coli* O157:H7. Food Microbiol..

[67] Zhang S., Liang X., Gadd G. M., Zhao Q. (2019). Advanced Titanium Dioxide-Polytetrafluorethylene
(TiO_2_-PTFE) Nanocomposite Coatings on Stainless Steel Surfaces
with Antibacterial and Anti-Corrosion Properties. Appl. Surf. Sci..

[68] Zhou N., López-Puente V., Wang Q., Polavarapu L., Pastoriza-Santos I., Xu Q.-H. (2015). Plasmon-Enhanced Light Harvesting:
Applications in Enhanced Photocatalysis, Photodynamic Therapy and
Photovoltaics. RSC Adv..

[69] Din M. I., Khalid R., Hussain Z. (2018). Minireview:
Silver-Doped Titanium
Dioxide and Silver-Doped Zinc Oxide Photocatalysts. Anal. Lett..

[70] Liu Z., Chen Z., Xie H., Cui Y., Feng H.-J., Hua K. (2023). The Effect of Electron Transfer Channel on UV-Independent Antibacterial
Activity of Ag^+^ Implanted TiO_2_. Appl. Surf. Sci..

[71] Mazare A., Goldmann W. H., Kocak E., Osuagwu B., Qin S., Cao R., Schmuki P. (2025). Silver-Loaded Titania-Based Metal–Organic
Frameworks
as a Platform for Silver Ion Release for Antibacterial Applications. Nano Lett..

[72] Shivaram A., Bose S., Bandyopadhyay A. (2017). Understanding
Long-Term Silver Release
from Surface Modified Porous Titanium Implants. Acta Biomater..

[73] Hou X., Ma H., Liu F., Deng J., Ai Y., Zhao X., Mao D., Li D., Liao B. (2015). Synthesis
of Ag Ion-Implanted TiO_2_ Thin Films for Antibacterial Application
and Photocatalytic
Performance. J. Hazard. Mater..

[74] Ortega F., Sobral P., Jios J. L., Arce V. B., García M. A. (2022). Starch
Nanocomposite Films: Migration Studies of Nanoparticles to Food Simulants
and Bio-Disintegration in Soil. Polymers.

[75] Ponsanti K., Tangnorawich B., Ngernyuang N., Pechyen C. (2020). A Flower Shape-Green
Synthesis and Characterization of Silver Nanoparticles (AgNPs) with
Different Starch as a Reducing Agent. J. Mater.
Res. Technol..

[76] Schaumann G. E., Philippe A., Bundschuh M., Metreveli G., Klitzke S., Rakcheev D., Grün A., Kumahor S. K., Kühn M., Baumann T., Lang F., Manz W., Schulz R., Vogel H.-J. (2015). Understanding the
Fate and Biological Effects of Ag- and TiO_2_-Nanoparticles
in the Environment: The Quest for Advanced Analytics and Interdisciplinary
Concepts. Sci. Total Environ..

[77] Hammad A. B. A., Al-esnawy A. A., Mansour A. M., El Nahrawy A. M. (2023). Synthesis
and Characterization of Chitosan-Corn Starch-SiO_2_/Silver
Eco-Nanocomposites: Exploring Optoelectronic and Antibacterial Potential. Int. J. Biol. Macromol..

[78] Nguyen M. T., Ha P. T., Huong
Le T. T., Bui H. G., Phan K. S., Chu N. H., Trang Mai T. T., Thi Ung T. D., Thi Le A. T., Hoang P. H. (2025). A Floatable
TiO_2_ – Ag Photocatalyst
Enables Effective Antibiotic Degradation and Pathogen Growth Control. RSC Adv..

[79] Sboui M., Alamry K. A., Al-Ghamdi Y. O., Subramanian B., Swaminathan M., Hussein M. A. (2025). Simple and Green
Fabrication of an
Ag-Ag_2_S/TiO_2_/Cellulose Biocomposite Film with
Enhanced Photocatalytic and Antibacterial Activity. RSC Adv..

[80] Kanakaraju D., Joseph D. N., Lim Y. C., Vincent M. (2025). Rapid Green Synthesis
of Ag-TiO_2_ Nanocomposites via Microwave Irradiation for
Water Treatment: Dual Action on Dyes and Bacteria. Asian J. Chem..

[81] Bao S., Sun S., Li L., Xu L. (2023). Synthesis and Antibacterial Activities
of Ag-TiO_2_/ZIF-8. Front. Bioeng.
Biotechnol..

[82] Singha S. K., Hoque S. M., Das H., Alim M. A. (2023). Evaluation of Chitosan-Ag/TiO_2_ Nanocomposite
for the Enhancement of Shelf Life of Chili
and Banana Fruits. Heliyon.

